# Integration-free induced pluripotent stem cells from three endangered Southeast Asian non-human primate species

**DOI:** 10.1038/s41598-023-50510-9

**Published:** 2024-01-29

**Authors:** Qiuye Bao, Nicole Liling Tay, Christina Yingyan Lim, Delia Hwee Hoon Chua, Su Keyau Kee, Mahesh Choolani, Yuin-Han Loh, Soon Chye Ng, Chou Chai

**Affiliations:** 1 Institute of Molecular and Cell Biology-Endangered Species Conservation By Assisted Reproduction (IMCB-ESCAR) Joint Laboratory, Agency for Science, Technology, and Research (A*STAR), 61 Biopolis Drive, Singapore, 138673 Singapore; 2 Department of Obstetrics and Gynaecology, Yong Loo Lin School of Medicine, National University of Singapore, Singapore, 119074 Singapore; 3 Mandai Wildlife Group, 80 Mandai Lake Road, Singapore, 729826 Singapore; 4 Cytogenetics Laboratory, Department of Pathology, Singapore General Hospital, 20 College Road, Singapore, 169856 Singapore; 5 Department of Biological Sciences, National University of Singapore, Singapore, 117543 Singapore; 6 Department of Physiology, Yong Loo Lin School of Medicine, National University of Singapore, Singapore, 117593 Singapore; 7 NUS Graduate School for Integrative Sciences and Engineering, National University of Singapore, 28 Medical Drive, Singapore, 117456 Singapore; 8 Sincere Healthcare Group, 8 Sinaran Drive, Singapore, 307470 Singapore; 9 Lee Kong Chian School of Medicine, Nanyang Technological University, 11 Mandalay Road, Singapore, 308232 Singapore

**Keywords:** Reprogramming, Induced pluripotent stem cells, Conservation biology

## Abstract

Advanced molecular and cellular technologies provide promising tools for wildlife and biodiversity conservation. Induced pluripotent stem cell (iPSC) technology offers an easily accessible and infinite source of pluripotent stem cells, and have been derived from many threatened wildlife species. This paper describes the first successful integration-free reprogramming of adult somatic cells to iPSCs, and their differentiation, from three endangered Southeast Asian primates: the Celebes Crested Macaque (*Macaca nigra*), the Lar Gibbon (*Hylobates lar*), and the Siamang (*Symphalangus syndactylus*). iPSCs were also generated from the Proboscis Monkey (*Nasalis larvatus*). Differences in mechanisms could elicit new discoveries regarding primate evolution and development. iPSCs from endangered species provides a safety net in conservation efforts and allows for sustainable sampling for research and conservation, all while providing a platform for the development of further in vitro models of disease.

## Introduction

We are currently in the midst of the Holocene extinction, the Earth’s sixth mass extinction crisis, this time directly caused by human activities^1^. Biodiversity conservation strategies have traditionally revolved around habitat preservation, maintenance and propagation of assurance populations, and assisted reproductive technologies (ART)^2^. These solutions naturally have their limitations, and it is therefore necessary to consider the benefits that advanced molecular and cellular technologies can bring to the future of conservation.

Induced pluripotent stem cells (iPSCs) were first generated in 2006^3^ by the reprogramming of adult somatic cells into pluripotent stem cells (PSCs). These cells have been shown to be similar to embryonic stem cells (ESCs) isolated from the inner cell mass (ICM) of a developing blastocyst^4^, capable of self-renewal and differentiation into any cell type of the primary germ layers. As iPSCs can be generated from almost any adult somatic cell type, regardless of donor age or gender, and does not require the destruction of an embryo, they offer an ethically acceptable, easily accessible, and infinite source of PSCs.

Early methods of iPSC generation involved integrating retroviral transduction of Yamanaka factors (*SOX2*, *OCT4*, *c-MYC*, *KLF4*) into the genome; this and other integrating approaches since developed are often efficient. However, they remain unsafe for therapeutic applications, due to the permanent genetic modifications or scarring of the genome which could result in genomic instability or insertional mutagenesis, which in turn may lead to tumorigenesis^5^. This led to the development of transgene integration-free approaches, which are often less efficient but more reproducible. These methods include integration-defective adenoviral^6,7^ or Sendai viral delivery^8–10^, transient episomal transfection^11–14^, RNA^15–18^ or protein delivery^19,20^, and chemical reprogramming^21–24^.

Integration-free approaches to reprogramming have revolutionized the fields of cellular and regenerative therapy, disease modelling, and drug discovery. Additionally, wildlife and environmental conservationists are looking to apply iPSC technology in attempts to rescue or revive critically endangered or extinct species, with increasing interest in the fields of in vitro gametogenesis^2,25,26^. Furthermore, the ability of iPSCs to differentiate towards any tissue type can reduce consumer dependence on meats derived from domestic species, or exotic animal products derived from species threatened with extinction, which in turn could reduce the environmental impacts of commercial animal husbandry and poaching respectively^27^. Compared to ESCs, which minimally require the harvesting of an oocyte and/or the destruction of an embryo, both of which are difficult to come by in endangered species, iPSCs offer a source of PSCs that are both practical and less ethically-challenging.

Early reprogramming efforts focused on mice^3^ and humans^28^, but iPSCs have now been derived from many other threatened wildlife species^26,27^, mostly using Yamanaka factors to reprogram cell lines taken from adult tissue. These successes demonstrate how iPSCs could be used to retain genetic diversity in endangered species with low population numbers. Nevertheless, species-specific reprogramming mechanisms remain poorly understood with varying difficulties in the generation of stable transgene-free iPSCs^29^.

This paper describes the first successful integration-free reprogramming of adult somatic cells to iPSCs, and their differentiation, from three endangered Southeast Asian primates: the Celebes Crested Macaque (*Macaca nigra*) is a Critically Endangered macaque endemic to Sulawesi, the Lar Gibbon (*Hylobates lar*) is an Endangered gibbon that can be found in continental South East Asia and Sumatra, and the Siamang (*Symphalangus syndactylus*) is an Endangered gibbon that can be found in Sumatra and Peninsula Malaysia. iPSCs from a fourth endangered Southeast Asian primate, the Proboscis Monkey (*Nasalis larvatus*), an Endangered colobine endemic to Borneo, was also generated, but was not demonstrated to be vector-free. Reprogramming of these non-human primate (NHP) species using modified and/or adapted human reprogramming protocols have not yet been reported in the literature until now. Integration-free approaches were used so that the derived iPSC lines could be exploitable for future downstream conservation applications. Sendai viral^8^ and episomal plasmid^30^ techniques were utilised over others for their known reliability and efficacy—the limited availability of early passage cell lines and sampling opportunities from these endangered species necessitated that reprogramming efforts be easily and quickly optimizable.

## Results

### Generation of Celebes Crested Macaque (*Macaca nigra*) iPSC lines

**Figure 1:**
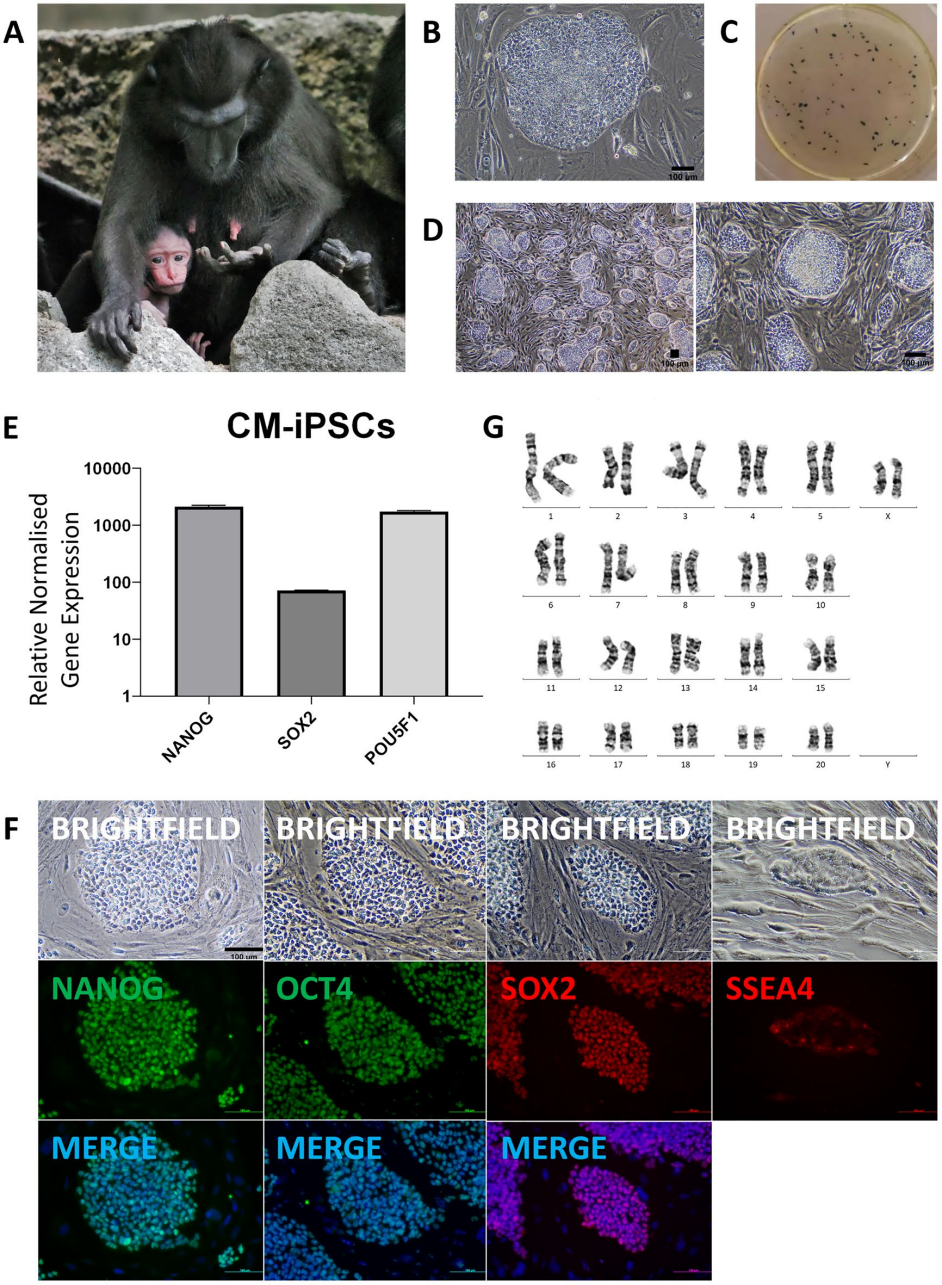
Generation of Celebes Crested Macaque (*Macaca nigra*) iPSC lines. (**A)** Celebes Crested Macaque (*Macaca nigra*). (**B)** Formation of CM-iPSC colonies at day 20 after transduction. (**C)** Alkaline phosphatase (AP) staining of CM-iPSCs corresponding to B. (**D)** CM-iPSC colonies at passage 4. (**E)** Quantitative RT-PCR analysis for the expression of endogenous pluripotency markers in CM-iPSCs normalized to *GAPDH* and controlled to source CM fibroblasts. **(F)** ICC staining confirming positive expression of pluripotency markers. (**G)** Karyotype of CM-iPSCs demonstrating 42 chromosomes, XX; 20 GTG-banded cells scored and analysed, 28 karyograms prepared.

To generate NHP PSCs for biobanking and research into pluripotency and differentiation, primary fibroblasts from a 21 year old female Celebes Crested Macaque (CM) (Fig. 1A) were reprogrammed via transduction with Sendai virus from the CytoTune™-iPS 2.0 kit containing human *KLF4*, *OCT3/4*, *SOX2*, and *c-MYC*. Additional lentiviral transduction of human *NANOG* alongside this primary reprogramming method did not improve reprogramming efficiency (0.12–0.14% for both methods).

Different media were tested to optimize the reprogramming efficiency. Fourteen days post-transduction in homemade embryonic stem cell (hES) media containing foetal bovine serum (FBS), primary colonies began forming. These developed after a further 6 days into colonies with similar morphologies to human ESCs, with a flat phenotype, smooth borders, and a high nuclear-to-cytoplasmic ratio, ready to be picked (Fig. 1B). hES medium was able to support a high reprogramming efficiency (0.12–0.14%, Fig. 1C), in contrast to the commercial serum-free medium mTeSR^TM^1 (0.016–0.017%) (Supplementary Fig. 1A). Culture and expansion of CM iPSCs (CM-iPSCs) could be supported on irradiated mouse embryonic fibroblasts (iMEF) in hES medium (Fig. 1D), mTeSR^TM^1, and a 50/50 mix of both medias. iPSCs were stable and could be passaged for more than 20 passages.

Transcript analysis revealed the increased expression of endogenous pluripotency markers at the mRNA level in CM-iPSCs (Fig. 1E, Supplementary Fig. 1B). When compared to human PSCs (H9), the expression of *OCT4* and *c-MYC* in CM-iPSCs was significantly lower and higher respectively (Supplementary Fig. 1C). Immunofluorescence (IF) staining confirmed positive expression of NANOG, SOX2, and OCT4 (Fig. 1F). However, the expression of SSEA-4 was not uniform throughout the colonies, with a low proportion of cells showing strong immunoreactivity. CM-iPSCs displayed a normal female karyotype with 20 matched pairs of autosomes and a single pair of X chromosomes (Fig. 1G), identical to that of their source fibroblasts (Supplementary Fig. 1D).

### CM-iPSCs are capable of differentiation into the three germ layers

**Figure 2:**
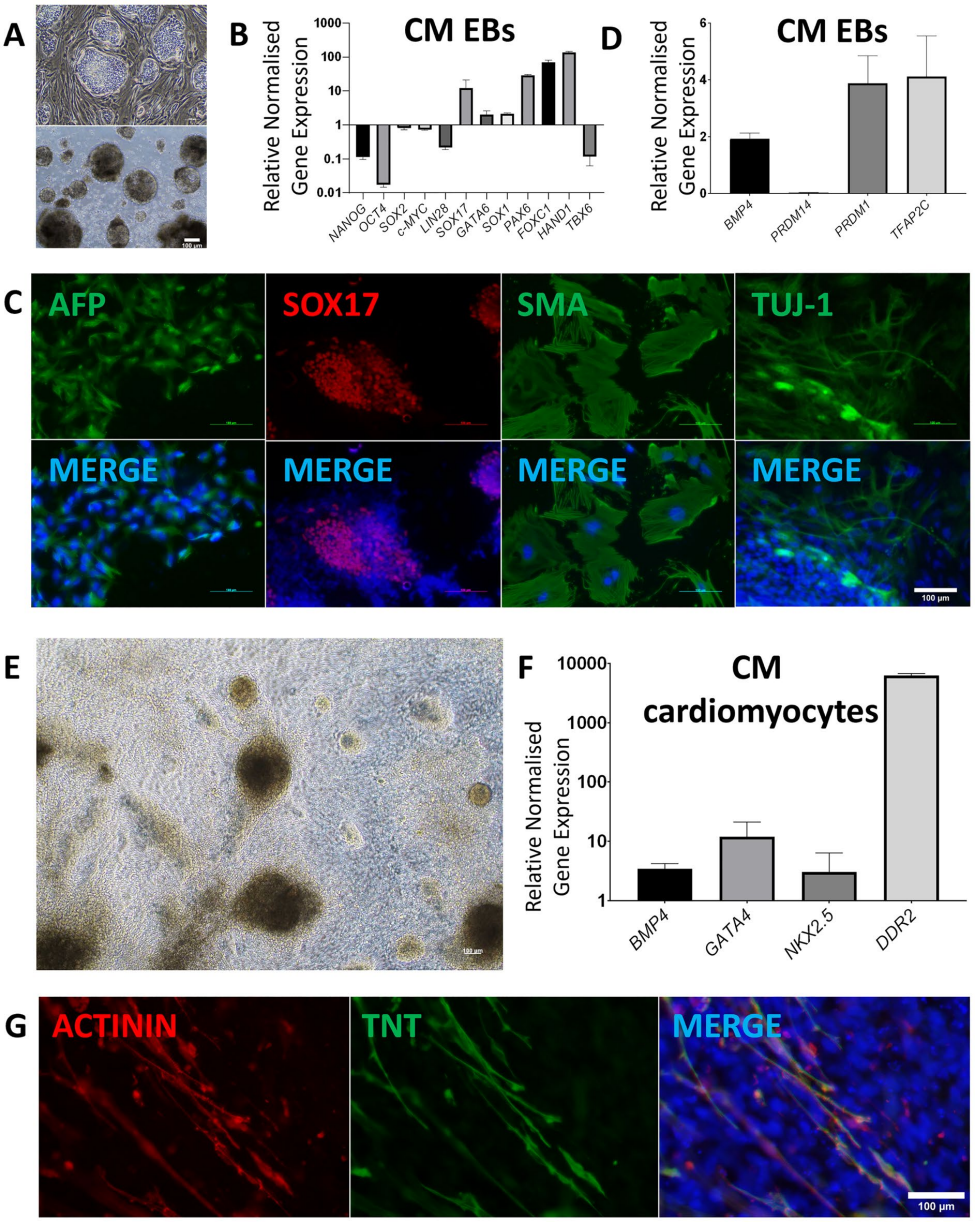
CM-iPSCs are capable of differentiation into the three germ layers and functional cardiomyocytes. **(A)** Differentiation of CM-iPSCs into EBs. (**B)** Quantitative RT-PCR analysis for the expression of pluripotency and germ layer markers in CM EBs normalized to *GAPDH* and controlled to source CM-iPSCs. (**C)** ICC staining of day 14 EBs confirming CM-iPSC differentiation into the three germ layers. (**D)** Quantitative RT-PCR analysis for the expression of PGC markers in CM EBs normalized to *GAPDH* and controlled to source CM-iPSCs. (**E)** Differentiation of CM-iPSCs into cardiomyocytes. (**F)** Quantitative RT-PCR analysis for the expression of cardiomyocyte markers in CM cardiomyocytes normalized to *GAPDH* and controlled to source CM-iPSCs. (**G)** ICC staining of CM cardiomyocytes confirming CM-iPSC differentiation into cardiomyocytes.

CM-iPSCs at different passages could form embryoid bodies (EBs) in either E5/6 medium or in hES medium without bFGF and hLIF (Fig. 2A). At Day 7, EBs were transferred into a gelatin-coated well, where they were allowed to attach and differentiate. When compared to the starting CM-iPSCs in transcript analysis, day 14 EBs demonstrated significantly increased expression of germ layer-specific markers for endoderm (*SOX17*, *GATA6*), mesoderm (*PAX6*, *FOXC1*), and ectoderm (*HAND1*, *TBX6*) (Fig. 2B). This was accompanied by a marked reduction in the expression of pluripotency markers *NANOG* and *OCT4*, with a less drastic reduction of *SOX2* and *c-MYC* expression. IF staining was able to detect and confirm the presence of germ layer-specific markers for endoderm (AFP, SOX17), mesoderm (SMA), and ectoderm (TUJ-1) (Fig. 2C).

Transcript analysis also revealed a 2 to fourfold increase in expression of primordial germ cell (PGC) markers *BMP4*, *PRDM1*, and *TFA2C* in EBs when compared against CM-iPSCs, suggesting possible PGC-differentiation in EBs formed from CM-iPSCs (Fig. 2D). However, *PRDM14* expression remained greater in CM-iPSCs than in EBs.

### CM-iPSCs are capable of differentiation into functional cardiomyocytes

A modified protocol was used to direct cardiomyocyte differentiation of CM-iPSCs in order to further validate their pluripotency and differentiation capacity. CM-iPSCs were induced towards mesodermal specification after 3 days of treatment with BMP4, Activin A, and FGF2, followed by IWP2 differentiation towards the cardiomyocyte lineage, before cardiomyocytes were finally obtained and maintained in insulin-containing medium^31^. By day 13, the differentiated cardiomyocytes demonstrated spontaneous contractility (Fig. 2E, Movie 1). Transcript analysis of day 30 cardiomyocytes demonstrated significantly increased expression of the cardiomyocyte markers *BMP4, GATA4, NKX2.5,* and *DDR2*, with *DDR2* showing an almost 1000-fold increase compared to CM-iPSCs (Fig. 2F). IF staining further confirmed the positive expression of cardiomyocyte markers cTnT and α-Actinin (Fig. 2G).

### Generation and differentiation of Lar Gibbon (*Hylobates lar*), Siamang (*Symphalangus syndactylus*), and Proboscis Monkey (*Nasalis larvatus*) iPSC lines

**Figure 3:**
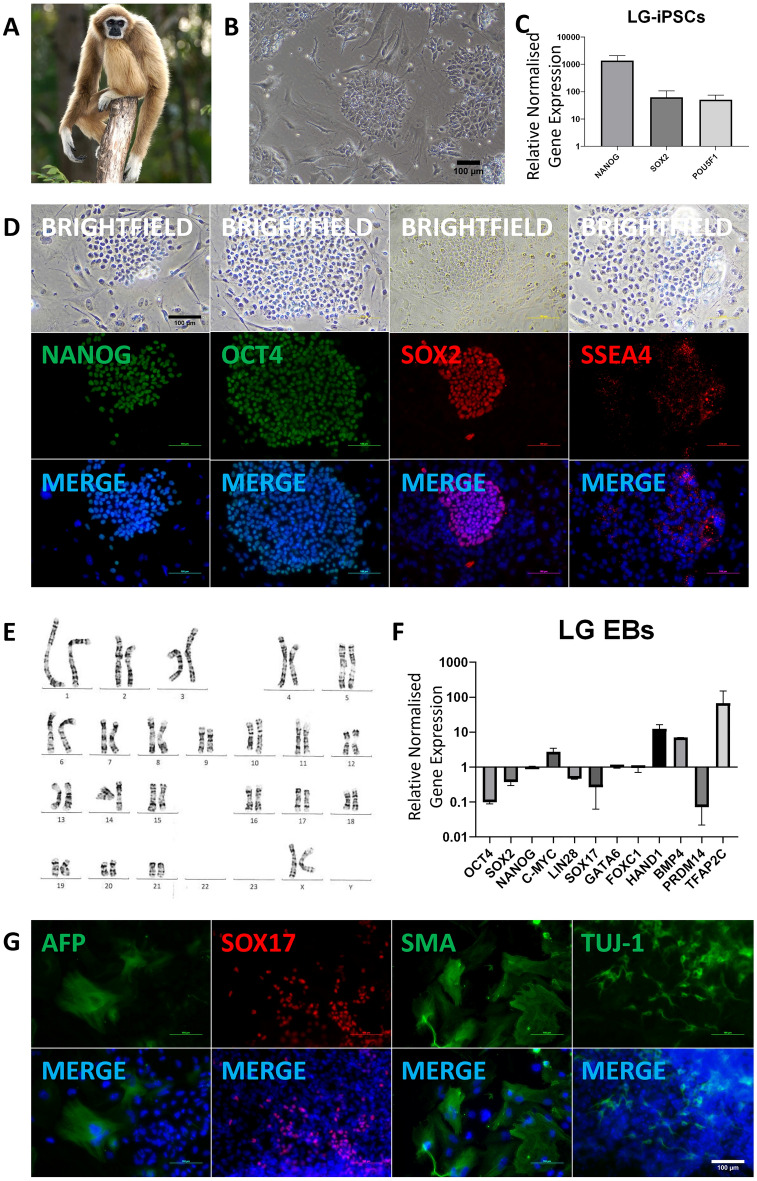
Generation and differentiation of Lar Gibbon (*Hylobates lar*) iPSC lines. **(A)** Lar Gibbon (*Hylobates lar*) **(B)** LG-iPSC colonies at passage 6. (**C)** Quantitative RT-PCR analysis for the expression of endogenous pluripotency markers in LG-iPSCs normalized to *GAPDH* and controlled to source LG fibroblasts. (**D)** ICC staining confirming positive expression of pluripotency markers in LG-iPSCs. (**E)** Karyotype of LG-iPSCs demonstrating 44 chromosomes, XX; 20 GTG-banded cells analysed, 20 karyograms prepared. (**F)** Quantitative RT-PCR analysis for the expression of pluripotency and germ layer markers in LG EBs normalized to *GAPDH* and controlled to source LG-iPSCs. (**G)** ICC staining of LG EBs confirming LG-iPSC differentiation into the three germ layers.

**Figure 4:**
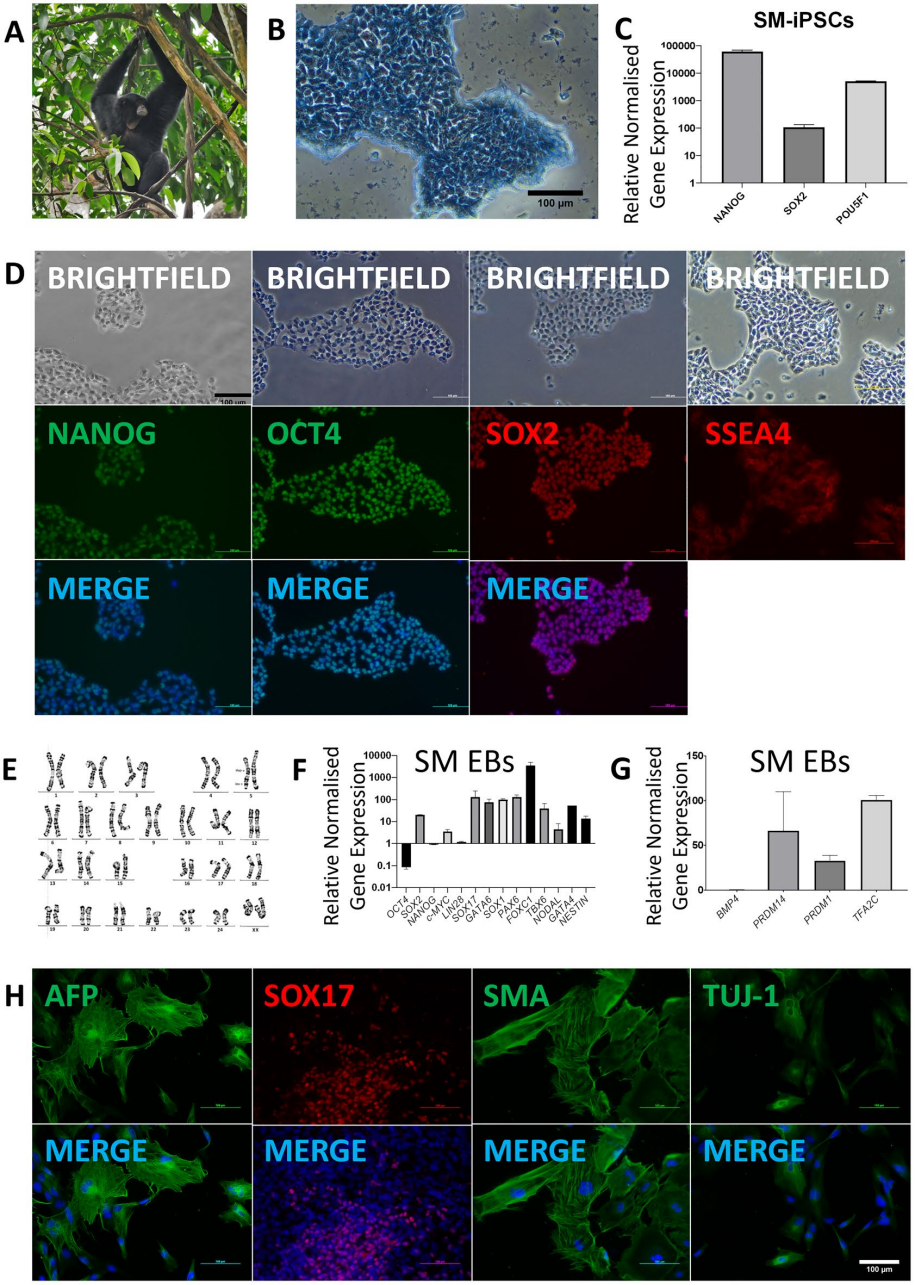
Generation and differentiation of Siamang (*Symphalangus syndactylus*) iPSC lines. (**A)** Siamang (*Symphalangus syndactylus*) **(B)** Alkaline phosphatase (AP) staining of SM-iPSC colonies at passage 9. (**C)** Quantitative RT-PCR analysis for the expression of endogenous pluripotency markers in SM-iPSCs normalized to *GAPDH* and controlled to source SM fibroblasts. (**D)** ICC staining confirming positive expression of pluripotency markers in SM-iPSCs. (**E)** Karyotype of SM-iPSCs demonstrating 50 chromosomes, XX; 20 GTG-banded cells analysed, 21 karyograms prepared. (**F)** Quantitative RT-PCR analysis for the expression of pluripotency and germ layer markers in SM EBs normalized to *GAPDH* and controlled to source SM-iPSCs. (**G)** Quantitative RT-PCR analysis for the expression of PGC markers in SM EBs normalized to *GAPDH* and controlled to source SM-iPSCs. (**H)** ICC staining of SM EBs confirming SM-iPSC differentiation into the three germ layers.

**Figure 5:**
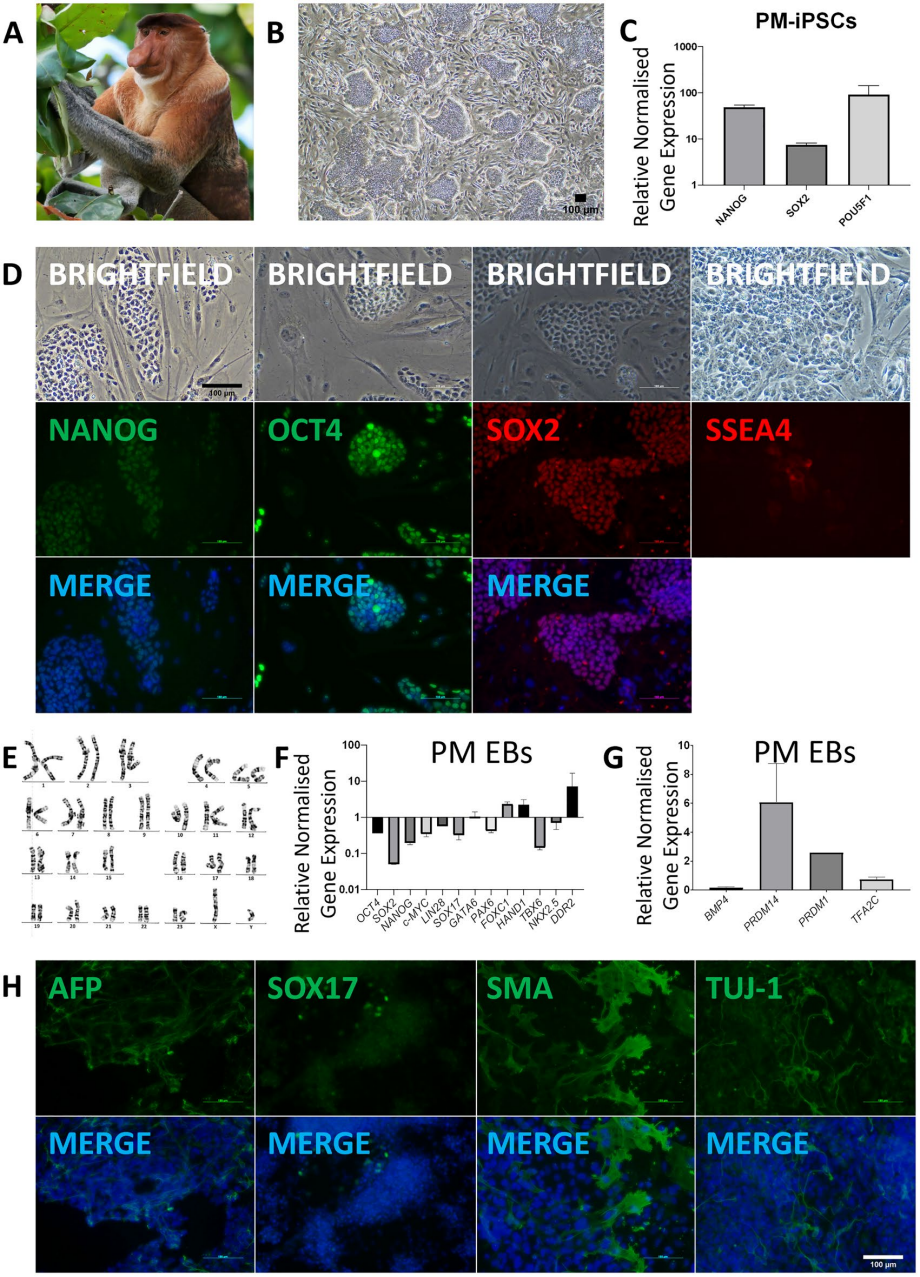
Generation and differentiation of Proboscis Monkey (*Nasalis larvatus*) iPSC lines. (**A)** Proboscis Monkey (*Nasalis larvatus*) **(B)** PM-iPSC colonies at passage 8. (**C)** Quantitative RT-PCR analysis for the expression of endogenous pluripotency markers in PM-iPSCs normalized to *GAPDH* and controlled to source PM fibroblasts. (**D)** ICC staining confirming positive expression of pluripotency markers in PM-iPSCs. (**E)** Karyotype of PM-iPSCs demonstrating 48 chromosomes, XY; 20 GTG-banded cells scored and analysed, 20 karyograms prepared. (**F)** Quantitative RT-PCR analysis for the expression of pluripotency and germ layer markers in PM EBs normalized to *GAPDH* and controlled to source PM-iPSCs. (**G)** Quantitative RT-PCR analysis for the expression of PGC markers in CM EBs normalized to GAPDH and controlled to source CM-iPSCs. (**H)** ICC staining of PM EBs confirming PM-iPSC differentiation into the three germ layers.

To generate further NHP PSCs for biobanking and research purposes, primary fibroblasts from a 6 year old female Lar Gibbon (LG) (Fig. 3A) were reprogrammed via transduction with Sendai virus from the CytoTune™-iPS 2.0 kit, while primary fibroblasts from a 4 month old female Siamang (SM) (Fig. 4A) and a 28 year old male Proboscis Monkey (PM) (Fig. 5A) were reprogrammed via transfection with episomal plasmids containing the Yamanaka factors. iPSC colonies began forming 1–2 weeks after transfection or transduction, with different morphologies between the species but all with sharp edges and a high nuclear-cytoplasmic ratio. Reprogramming efficiencies for LG, SM, and PM were > 1%, 0.19%, and > 1% respectively. Lar Gibbon iPSCs (LG-iPSCs) were maintained and expanded on iMEF (Fig. 3B), and demonstrated optimal growth in mTeSR^TM^Plus (Supplementary Fig. 2A). Siamang iPSCs (SM-iPSCs) were maintained and expanded on Matrigel®-coated wells in mTeSR^TM^1 (Fig. 4B). Proboscis Monkey iPSCs (PM-iPSCs) were maintained and expanded on iMEF (Fig. 5B), and demonstrated optimal growth in StemMACS™ iPS-Brew XF (Supplementary Fig. 4A). iPSCs were stable and could be passaged for more than 20 passages.

Transcript analysis demonstrated significant upregulation of endogenous pluripotency markers in LG-iPSCs, SM-iPSCs, and PM-iPSCs when compared to their source fibroblasts (Figs. 3C, 4C, 5C, Supplementary Figs. 2B, 3A, 4B). Correspondingly, IF staining demonstrated the positive expression of pluripotency markers in LG-iPSCs, SM-iPSCs, and PM-iPSCs (Figs. 3D, 4D, 5D). LG-iPSCs displayed a normal male karyotype with 21 autosomal pairs and an X chromosome pair (Fig. 3E), identical to reference karyograms^32^. On the other hand, SM-iPSCs displayed a female karyotype with 25 pairs of chromosomes, with 24 autosomal pairs and an X chromosome pair. Chromosome 5 appeared to carry two rearrangements including an interstitial duplication of the p-arm and a paracentric inversion involving the distal segment of the q-arm (Fig. 4E). However, the original Siamang fibroblasts demonstrated an identical karyotype (Supplementary Fig. 3B), suggesting that the reprogramming process was not the cause of the abnormality. PM-iPSCs displayed a normal male karyotype with 23 autosomal pairs and an X and Y gonosome (Fig. 5E), identical to their original fibroblasts (Supplementary Fig. 4C).

Through EB formation, LG-iPSCs, SM-iPSCs, and PM-iPSCs were able to differentiate into the three germ layers. When compared with their starting iPSCs in transcript analysis, EBs from these species demonstrated an increased expression of germ layer-specific and PGC-specific markers and reduced expression of pluripotency markers, with the exception of *SOX2* being more highly expressed in Siamang EBs than in SM-iPSCs (Figs. 3F, 4F, 4G, 5F, 5G). IF staining demonstrated germ layer-specific markers for endoderm (AFP, SOX17), mesoderm (SMA), and ectoderm (TUJ-1) (Figs. 3G, 4H, 5H).

### CM-iPSCs, LG-iPSCs, and SM-iPSCs are integration-free

**Figure 6:**
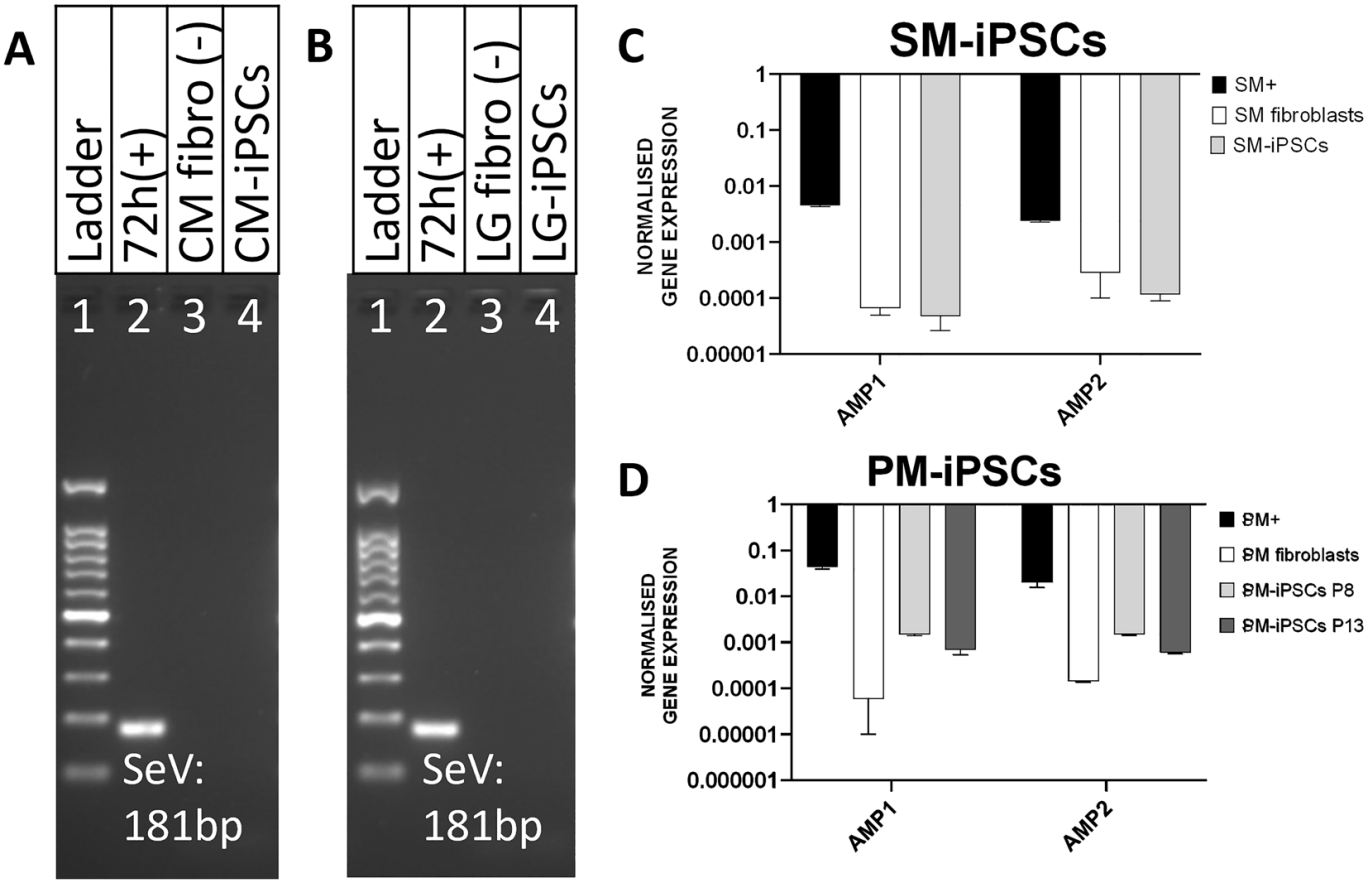
Demonstration of transgene-free iPSC lines. (**A**) CM-iPSCs: gel electrophoresis of PCR amplified products using primers to detect the Sendai viral genome and transgenes. (1) 100 bp DNA ladder; (2) CM fibroblasts 72 h post-transduction (positive control) demonstrating the 181 bp fragment from the Sendai viral genome; (3) CM fibroblasts (negative control); (4) CM-iPSCs demonstrating lack of detection of Sendai viral genome. (**B**) LG-iPSCs: gel electrophoresis of PCR amplified products using primers to detect the Sendai viral genome and transgenes. (1) 100 bp DNA ladder; (2) LG fibroblasts 72 h post-transduction (positive control) demonstrating the 181 bp fragment from the Sendai viral genome; (3) LG fibroblasts (negative control); (4) LG-iPSCs demonstrating lack of detection of Sendai viral genome. (**C**) SM-iPSCs: Quantitative RT-PCR analysis confirming SM-iPSCs’ lack of expression of ampicillin-resistance genes found in the backbone of the reprogramming plasmids normalized to *GAPDH*; SM + refers to SM fibroblasts 1 week post-transfection (positive control). (**D**) PM-iPSCs: Quantitative RT-PCR analysis unable to confirm PM-iPSCs’ lack of expression of ampicillin-resistance genes found in the backbone of the reprogramming plasmids normalized to *GAPDH*; PM + refers to PM fibroblasts 1 week post-transfection (positive control).

Using primers designed to detect the Sendai viral genome and transgenes, PCR-amplified products were visualized on gel electrophoresis, demonstrating the lack of Sendai viral transgenes in CM-iPSCs and LG-iPSCs (Figs. 6A, 6B), indicating that these iPSC lines are integration-free. Similarly, using primers designed to detect the ampicillin-resistance genes found in the backbone of the episomal plasmids, transcript analysis demonstrated the lack of expression of the reprogramming vectors in SM-iPSCs (Fig. 6C), indicating that this iPSC line is integration-free. However, transcript analysis using the same primers were able to detect a moderate amount of expression of the reprogramming vectors in PM-iPSCs (Fig. 6D), suggesting that this iPSC line is not vector-free; although expression levels appear to decrease with increasing passages.

## Discussion

In the midst of the current extinction crisis, NHP species as our closest relatives have not been spared, with 60% of all primate species classified as threatened with extinction by the International Union for Conservation of Nature (IUCN)^33^. Efforts to conserve these species are justified in the light of the value that they provide ecologically, economically, culturally, and intellectually^34,35^.

As humans and these NHP species shared a common ancestor 25 million years ago^36^, it was hypothesized that mechanisms of reprogramming for iPSCs would be conserved amongst these primates. Previous reprogramming efforts in NHP species used mostly integrating retroviral or lentiviral methods^37–44^, with Sendai viral or episomal methods only being performed successfully in two NHP species^45,46^. Therefore, in attempting to generate transgene-free iPSCs from these NHPs, these two efficient and reliable methods commonly used in the reprogramming of human cells were used. CM fibroblasts were able to be reprogrammed via Sendai viral (Fig. 1) and episomal methods (data not included); however, PM fibroblasts were only successfully reprogrammed via episomal methods (Fig. 5). (LG and PM fibroblasts were subjected only to reprogramming via Sendai viral and episomal methods respectively.) This suggests inherent differences in the transcriptional requirements governing reprogramming to pluripotency across different species. Such differences may extend to the differentiation potential of the iPSCs as well. Similarly, despite IF data demonstrating the successful EB formation from PM-iPSCs (Fig. 4), transcriptomic analysis remained unconvincing for the expression of endoderm genes. These unexpected differences in preferred reprogramming and differentiation methods between the species suggests that cellular mechanisms may not be entirely conserved amongst the primates, and further in-depth studies into these differing mechanisms could elicit new and interesting findings regarding the evolution and development of these distinct species. At a more practical level, it is also clear that even different species in the same phylogenetic order may require different media in the generation of iPSCs.

Similarly, cardiomyocyte differentiation amongst the different iPSCs were also variable. Three separate protocols were attempted for the iPSCs, with variable outcomes. CM-iPSCs were able to efficiently differentiate to cardiomyocytes with spontaneous contractility (Fig. 2E,F,G). On the other hand, IF of cardiomyocytes differentiated from PM-iPSCs and SM-iPSCs showed the presence of cTnT and α-Actinin (Supplementary Fig. 3C, 4D), but failed to display any spontaneous contractility, suggesting that the applied differentiation and/or culture conditions were not optimal for these species. Further optimization would help to refine the protocol and expand the possibilities for the use of this model in the study of molecular and cellular mechanisms behind myocardial pathologies. Cardiovascular disease, in particular idiopathic myocardial fibrosis, is a major cause of mortality in both young and geriatric Great Apes managed in captivity^47^. This presents a major threat not only to the individual animals but also can negatively impact genetic diversity within a captive population. Despite this, current understanding of the epidemiology, diagnosis, and treatment of this and associated conditions remains poor. There is a critical need to investigate and understand the mechanisms behind this disease. With poor accessibilities and feasibilities in carrying out prospective and/or retrospective studies on both captive and wild Great Apes, perhaps a solution exists in in vitro models.

Preventing the extinction event of any species is the main goal of conservation, and iPSCs have the potential to play a major role in these efforts, alongside broader efforts such as cellular agriculture and the production of typically-poached animal products. The successful transgene-free generation of iPSCs from three endangered Southeast Asian primates, as presented here (Fig. 6A,B,C), adds to the existing examples of how iPSCs can provide an essential safety net in these conservation efforts^40,44,48–50^. Unfortunately, it was not possible to conclusively demonstrate vector-free iPSC lines in PM-iPSCs, although plasmid expression might continue to decrease with even later passages (Fig. 6D). While it is generally accepted that episomal vectors are removed spontaneously from cells during cell division^30^, retention of episomal plasmid sequences can occur^51^. In future, strategies involving negative selection of cells demonstrating such episomal retention could be employed to generate safe iPSCs for translational applications^52^.

Cryopreservation of tissue, somatic cells, and gametes have been used as an approach to safeguard valuable genetic material of species and individuals^53^. This genome resource banking has included cultured somatic cells like fibroblasts, which are limited in availability due to eventual senescence and depletion. In contrast, iPSCs have the potential to provide a self-renewing and inexhaustible resource of genetic material from endangered species and individuals that may no longer even be alive, allowing for sustainable sampling for research and conservation, with the possibility of expanding the genetic diversity available. Cryopreservation of iPSCs and making them available would assist research in physiology, phylogeography, taxonomy, relatedness, and genetic diversity, all of which would contribute to decision-making in the management of ex situ and in situ wildlife populations.

Nevertheless, it is important to recognize that biobanking is not a substitute for species conservation, and that closely linked to these efforts are ART. iPSC-derived mice and pigs have been achieved via tetraploid complementation and/or nuclear transfer at low efficiencies^54–58^, but have not yet been demonstrated in endangered wildlife species, possibly due to the poor availability of donor oocytes or embryos; although the possibility of using a related domestic species remains^59–66^. Creation of functional iPSC-derived gametes as demonstrated in rodents^67–70^ is also looking to be a viable solution in the preservation of critically endangered species like the Northern White Rhinoceros^71^. This ability to produce an inexhaustible supply of haploid gametes would provide an endless resource for basic research into gamete and embryo physiology of endangered wildlife species. In addition, iPSC-derived gametes from frozen cells previously sampled from dead individuals would enable the infusion of genetic diversity into a limited population.

These examples illustrate the potential value of iPSCs in conservation efforts. However, no wildlife species is currently being managed using cellular- or embryo-based approaches, which could be due to insufficient understanding of reprogramming, reproductive physiology, and funding. Furthermore, due caution must be taken as reprogramming can lead to chromosome aberrations, mutations, and epigenetic abnormalities^72^. Therefore, in-depth research into the mechanisms of reprogramming and fidelity measures such as karyotyping must be undertaken to ensure the production of safe iPSCs for downstream applications.

## Materials and methods

### Ethics statement

This project has been approved by the MWG Research Panel under the project code MWG230104. No animals were harmed in the preparation of this manuscript.

### Resource availability

This study generated new unique resources. Further information and requests for resources and reagents should be directed to the corresponding author.

### Access to skin samples

The IMCB-ESCAR laboratory is part of the Mandai Wildlife Group, which is the steward of Mandai Wildlife Reserve, home to Singapore Zoo, Night Safari, River Wonders, and Bird Paradise. Animals either died of natural causes or were humanely euthanised due to medical reasons. Skin samples were obtained from only one donor animal per species.

### Derivation and culture of NHP primary fibroblasts

At post-mortem, the animals’ skin was cleaned with 70% ethanol and shaved before aseptic surgical preparation of the area of interest. A sterile scalpel blade was used to harvest a 3 cm x 3 cm sample of full-thickness skin. In sterile conditions, any remaining fur, fat, and epidermis were removed from the skin sample, and the sample was cut into smaller pieces. After washing in PBS, the pieces were incubated in DMEM, 1X Antibiotic–Antimycotic, and 0.5 mg/ml Fungin™, at room temperature for 30 min. After further washing in PBS, the pieces were placed in a 0.1% gelatin-coated sterile tissue culture dishes with media containing DMEM, 20% FBS, 1X MEM non-essential amino acids (NEAA), 1X Penicillin–Streptomycin, and 2 mM L-glutamine, and incubated at 37 degrees Celsius and 5% CO_2_. When confluent, fibroblasts were passaged with 0.25% trypsin and maintained on 0.1% gelatin-coated sterile tissue culture dishes with fibroblast media (FM) containing DMEM, 10% FBS, 1X MEM NEAA, 100 µM β-mercaptoethanol, 1X Penicillin–Streptomycin, and 2 mM L-glutamine. For efficient reprogramming, fibroblasts were transduced or transfected at passage 5 or earlier.

### Derivation and culture of Crested Macaque iPSCs

250 k Crested Macaque fibroblasts were seeded onto 0.1% gelatin-coated 3 cm dishes and cultured in FM. 24 h later (Day 0), fibroblasts were transduced with Sendai virus from the CytoTune™-iPS 2.0 kit (Thermo Fisher Scientific, A16517) containing human *KLF4*, *OCT3/4*, *SOX2*, and *c-MYC*, in 1 ml of FM. On Day 1, the media was changed to FM containing 10 ng/ml bFGF and 0.4 mM sodium butyrate, and was refreshed daily. On Day 6, cells were passaged and split 1:4 into 6-well plates coated with irradiated CF1 mouse embryonic fibroblast (iMEF) feeder layers or Matrigel®, and cultured in FM with bFGF. On Day 7, the media was changed to homemade embryonic stem cell (hES) or mTeSR^TM^1 media, and refreshed daily. hES media contained DMEM, 15% ESC-screened FBS, 1X MEM NEAA, 100 µM β-mercaptoethanol, 1X Penicillin–Streptomycin, 2 mM L-glutamine, 10 ng/ml bFGF, and 10 ng/ml hLIF. mTeSR^TM^1 media contained 0.5 mM sodium butyrate until Day 11. Reprogramming efficiency was calculated at Day 20, and was derived from dividing the resultant number of colonies as visualised on alkaline phosphatase (AP) staining by the starting cell number and an estimated transduction efficiency of 90%. iPSC colonies were picked from Day 20, and expanded and subsequently maintained in 6-well plates seeded with feeders in hES media. iPSCs were stable and could be passaged for more than 20 passages. Seven iPSC lines were generated and one was used for further analysis.

### Derivation and culture of Lar Gibbon iPSCs

250 k Lar Gibbon fibroblasts were seeded onto 0.1% gelatin-coated 3 cm dishes and cultured in FM. 24 h later (Day 0), fibroblasts were transduced with Sendai virus from the CytoTune™-iPS 2.0 kit in 1 ml of FM. On Day 1, the media was changed to FM containing 10 ng/ml bFGF and 0.4 mM sodium butyrate, and was refreshed daily. On Day 6, cells were passaged and split 1:4 into 6-well plates coated with iMEF feeder layers or Matrigel®, and cultured in FM with bFGF. On Day 7, the media was changed to hES or mTeSR^TM^Plus (STEMCELL Technologies, #100–0276) media, and refreshed daily. mTeSR^TM^Plus media contained 0.5 mM sodium butyrate until Day 11. Reprogramming efficiency was calculated at Day 22, and was derived from dividing the resultant number of colonies as visualised on alkaline phosphatase (AP) staining by the starting cell number and an estimated transduction efficiency of 90%. iPSC colonies were picked from Day 23, and expanded and subsequently maintained in 6-well plates seeded with feeders in mTeSR^TM^Plus. iPSCs were stable and could be passaged for more than 20 passages. Six iPSC lines were generated and one was used for further analysis.

### Derivation and culture of Siamang iPSCs

500 k Siamang fibroblasts were transfected with 2ug each of plasmids pCXLE-hUL, pCXLE-hSK, pCXLE-OCT3/4-p53shRNA, and pCXWB-EBNA-1 (Addgene #27,080, #27,078, #27,077, and #37,624 respectively). Electroporation was performed via the Neon™ transfection system (Thermo Fisher Scientific, MPK5000, MPK1025) at 1650 V, 10 ms, and 2–3 pulses. Transfected cells were seeded onto 0.1% gelatin-coated 6 cm dishes and cultured in FM (Day 0). 24 h later (Day 1), the media was changed to FM containing 10 ng/ml bFGF and 1 mM sodium butyrate, and was refreshed daily. On Day 7, cells were passaged and split 1:3 into 6-well plates coated with Geltrex™ and cultured in either mTeSR™ Plus or StemMACS™ iPS-Brew XF (Miltenyi Biotec, 130–104-368). Reprogramming efficiency was calculated at Day 13, and was derived from dividing the resultant number of colonies as visualised on alkaline phosphatase (AP) staining by the starting cell number and a transfection efficiency of 55.69% as determined by FACS analysis of pCXLE-EGFP (Addgene #27,082) transfected cells at day 2 under identical conditions. iPSC colonies were picked from Day 14, and expanded and subsequently maintained in 6-well plates coated with Geltrex™, in iPSC media. iPSCs were stable and could be passaged for more than 20 passages.

### Derivation and culture of Proboscis Monkey iPSCs

600 k Proboscis Monkey fibroblasts were transfected with 2ug each of plasmids pCXLE-hUL, pCXLE-hSK, pCXLE-OCT3/4-p53shRNA, and pCXWB-EBNA-1. Electroporation was performed via the Neon™ Transfection System at 1650 V, 10 ms, and 3 pulses. Transfected cells were seeded onto 0.1% gelatin-coated 6 cm dishes and cultured in FM without Penicillin–Streptomycin (Day 0). 24 h later (Day 1), the media was changed to FM containing 10 ng/ml bFGF. On Day 2, the media was changed to FM containing 10 ng/ml bFGF and 0.5 mM sodium butyrate, and was refreshed daily. On Day 7, cells were passaged and split 1:4 into 6-well plates coated with Matrigel® or Geltrex™, and cultured in a 1:1 ratio of FM and a variety of iPSC medias with 10 µM Y-27632. iPSC medias were either StemMACS™ iPS-Brew XF, PluriSTEM™ Human ES/iPS Cell Medium (MERCK, SCM130), or Essential 8™ Medium (Thermo Fisher Scientific, A1517001) with 0.5 mM sodium butyrate until and including Day 11. On Day 9, the media was changed to iPSC media only, and was refreshed every other day. iPSC colonies started forming by Day 13. Reprogramming efficiency was calculated at Day 20, and was derived from dividing the resultant number of colonies as visualised on alkaline phosphatase (AP) staining by the starting cell number and a transfection efficiency of 40.58% as determined by fluorescence-activated cell sorting (FACS) analysis of pCXLE-EGFP transfected cells at day 2 under identical conditions. iPSC colonies were picked from Day 21, and expanded and subsequently maintained in 6-well plates coated with Matrigel®, Geltrex™, or feeders, in iPSC media; different coatings were used to optimize for the best culture conditions. iPSCs were stable and could be passaged for more than 20 passages. Three iPSC lines were generated and one was used for further analysis.

### Karyotyping

Cells were treated overnight with 2.5 mg/ml 5-bromo-2-deoxyuridine and 10ug/ml colcemid. Cells were then dissociated with 0.5% trypsin–EDTA for 10 min, collected into a centrifuge tube, and treated with 5 ml hypotonic solution containing 75 mM KCl and 0.8% sodium citrate for 20 min. Cells were then fixed with a series of 3:1 methanol and acetic acid fixative. The cell suspension was then dropped onto clean wet slides and allowed to air dry, and placed in a 90 degree Celsius oven prior to staining. G-banding and chromosome analyses were performed in accordance with standard procedures. 20 cells were analyzed per sample. Reference karyograms were taken from the Atlas of Mammalian Chromosomes^32^.

### Embryoid body formation and differentiation into three germ layers

iPSCs were passaged with ReLeSR™ (STEMCELL Technologies, #100–0484) and split 1:3 into ultralow attachment 24-well plates. Cells were cultured in hES media without bFGF nor hLIF for 7 days. EBs formed were either maintained in suspension for another 7 days before being harvested for RNA extraction, or transferred to a 0.1% gelatin-coated plate for another 7 days before being fixed for immunocytochemical analysis.

### Differentiation into cardiomyocytes

iPSCs were differentiated into cardiomyocytes using a modified protocol^31^. iPSCs were seeded into Matrigel®-coated wells and cultured in their respective preferred iPSC media. 2 days later, the media was changed to RPMI 1640, B27 supplement without insulin, 10 ng/ml Activin A, 10 ng/ml BMP4, and 10 ng/ml bFGF. After 3 days, the media was changed to RPMI 1640, B27 supplement without insulin, with 3 µM IWP-2. 4 days later, the differentiated cells were maintained in RPMI 1640 containing B27 supplement with insulin.

### Quantitative RT-PCR

Total RNA was extracted using Monarch® Total RNA Miniprep Kit (New England BioLabs Inc., T2010) and reverse transcribed into cDNA with iScript™ Reverse Transcription Supermix (Bio-Rad, 1,708,840). qPCR was performed using KAPA SYBR® FAST qPCR Master Mix (2X) Universal (Kapa Biosystems, KK4618) with 2.5 ng cDNA with a primer concentration of 115 nM. Samples were run in duplicates and GAPDH was used as internal control. Primers are listed in Supplementary Table 1. To measure endogenous expression of pluripotency markers *OCT4*, *SOX2*, and *NANOG*, primers were designed to the 3’UTRs of the mRNA sequences. *OCT4* refers to *OCT4A*. Detection of Sendai viral reprogramming vectors was performed using primers described in the CytoTune™-iPS 2.0 kit protocol.

### Immunofluorescence staining

Attached cells were washed in PBS, fixed with 4% PFA for 10 min, and washed again. Blocking was performed at room temperature for 2 h; blocking buffer contained 2% BSA, 0.3% triton X-100, in PBS. Incubation with primary antibodies was performed at 4 degrees Celsius overnight, before washing with 0.2% tween-20 in PBS. Incubation with secondary antibodies and Hoechst was performed in the dark at room temperature for 1 h, before washing with PBS. Images were acquired on a fluorescence microscope. ImageJ was used to overlay images obtained from the fluorescence microscope, and to improve visualisation of the scale bars when necessary. Antibodies are listed in Supplementary Table 2.

### Supplementary Information

Supplementary Information 1.

Supplementary Video 1.

## Data Availability

The authors declare that the data supporting the findings of this study are available within the paper and its Supplementary Information files. Should any raw data files be needed in another format they are available from the corresponding author upon reasonable request.
