# Supplementary material for: Integration-free induced pluripotent stem cells from three endangered Southeast Asian non-human primate species
Source: Sci Rep. 2024 Jan 29;14:2391. doi: 10.1038/s41598-023-50510-9 (PMC10825216; doi:10.1038/s41598-023-50510-9)
Supplement: Supplementary file 1 — Supplementary Information 1. [file 41598_2023_50510_MOESM1_ESM.docx]

**SUPPLEMENTARY INFORMATION**

**INTEGRATION-FREE INDUCED PLURIPOTENT STEM CELLS FROM
THREE ENDANGERED SOUTHEAST ASIAN NON-HUMAN PRIMATE SPECIES**

**Qiuye Bao**

**Nicole Liling Tay**

Christina Yingyan Lim

Delia Hwee Hoon Chua

Kee Su Keyau

Yuin-Han Loh

Soon Chye Ng

Chou Chai

**
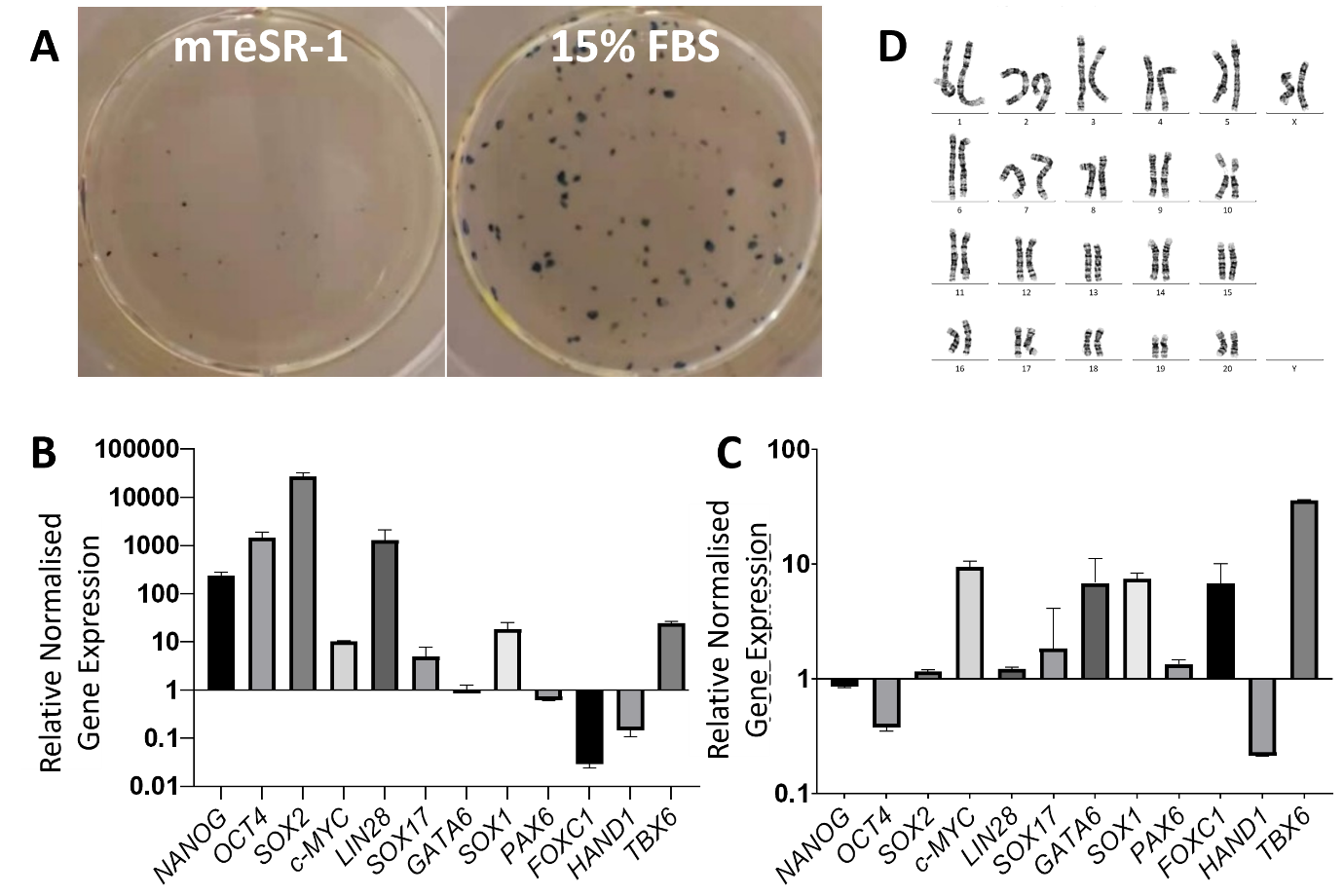
**

**Supplementary Figure 1: Reprogramming for CM-iPSCs. A.** AP staining of CM-iPSCs reprogrammed in mTeSR-1 or homemade media containing 15% FBS. **B.** Quantitative RT-PCR analysis for the expression of pluripotency and germ layer markers in CM-iPSCs normalized to *GAPDH* and controlled to source CM fibroblasts. **C.** Quantitative RT-PCR analysis for the expression of pluripotency and germ layer markers in CM-iPSCs normalized to *GAPDH* and controlled to source human PSCs (H9). **D.** Karyotype of CM fibroblasts demonstrating 42 chromosomes, XX.

**
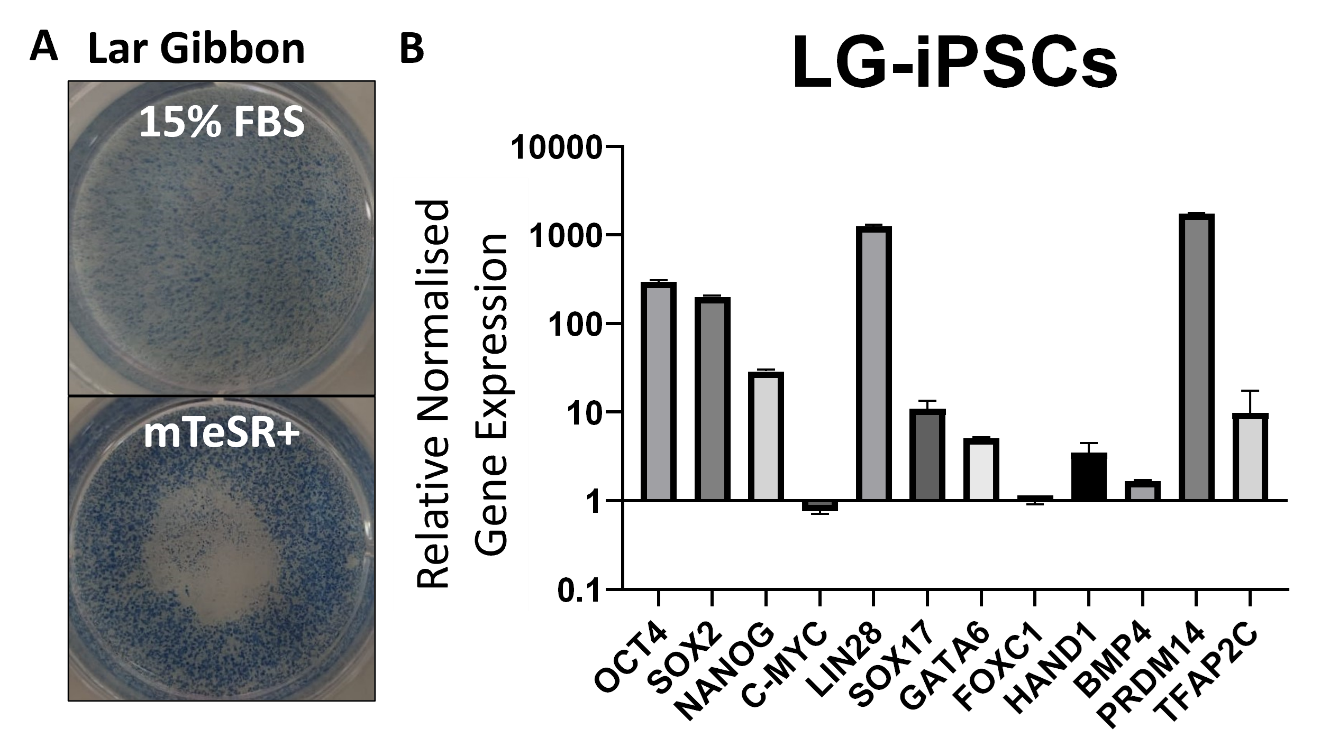
**

**Supplementary Figure 2: Reprogramming for LG-iPSCs**. **A.** AP staining of LG-iPSCs reprogrammed in different media. **B.** Quantitative RT-PCR analysis for the expression of pluripotency and germ layer markers in LG-iPSCs normalized to *GAPDH* and controlled to source LG fibroblasts.

**
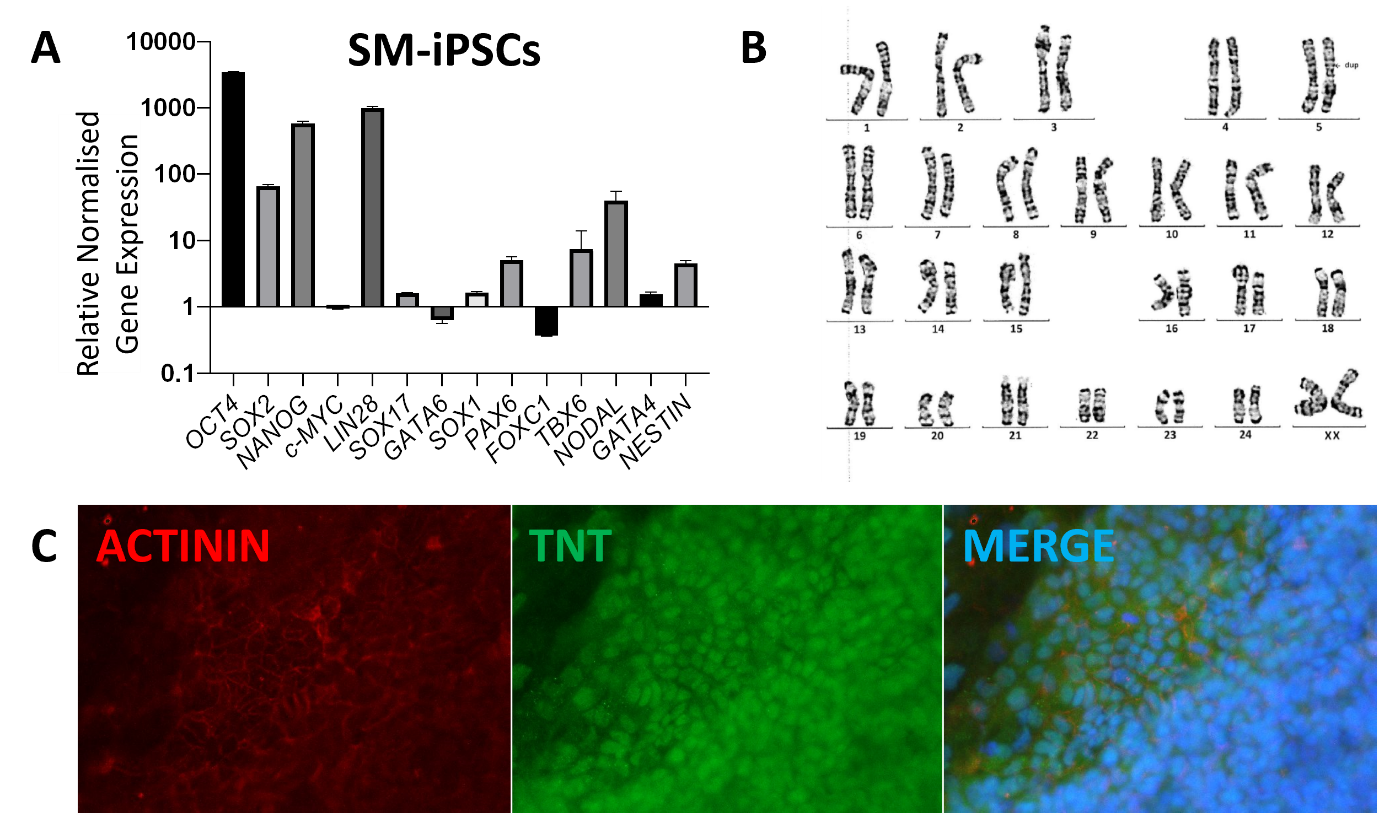
**

**Supplementary Figure 3: Reprogramming for and differentiation of SM-iPSCs**. **A.** Quantitative RT-PCR analysis for the expression of pluripotency and germ layer markers in SM-iPSCs normalized to *GAPDH* and controlled to source SM fibroblasts. **B.** Karyotype of SM fibroblasts demonstrating 50 chromosomes, XX. **C.** ICC staining of SM-cardiomyocytes.

**
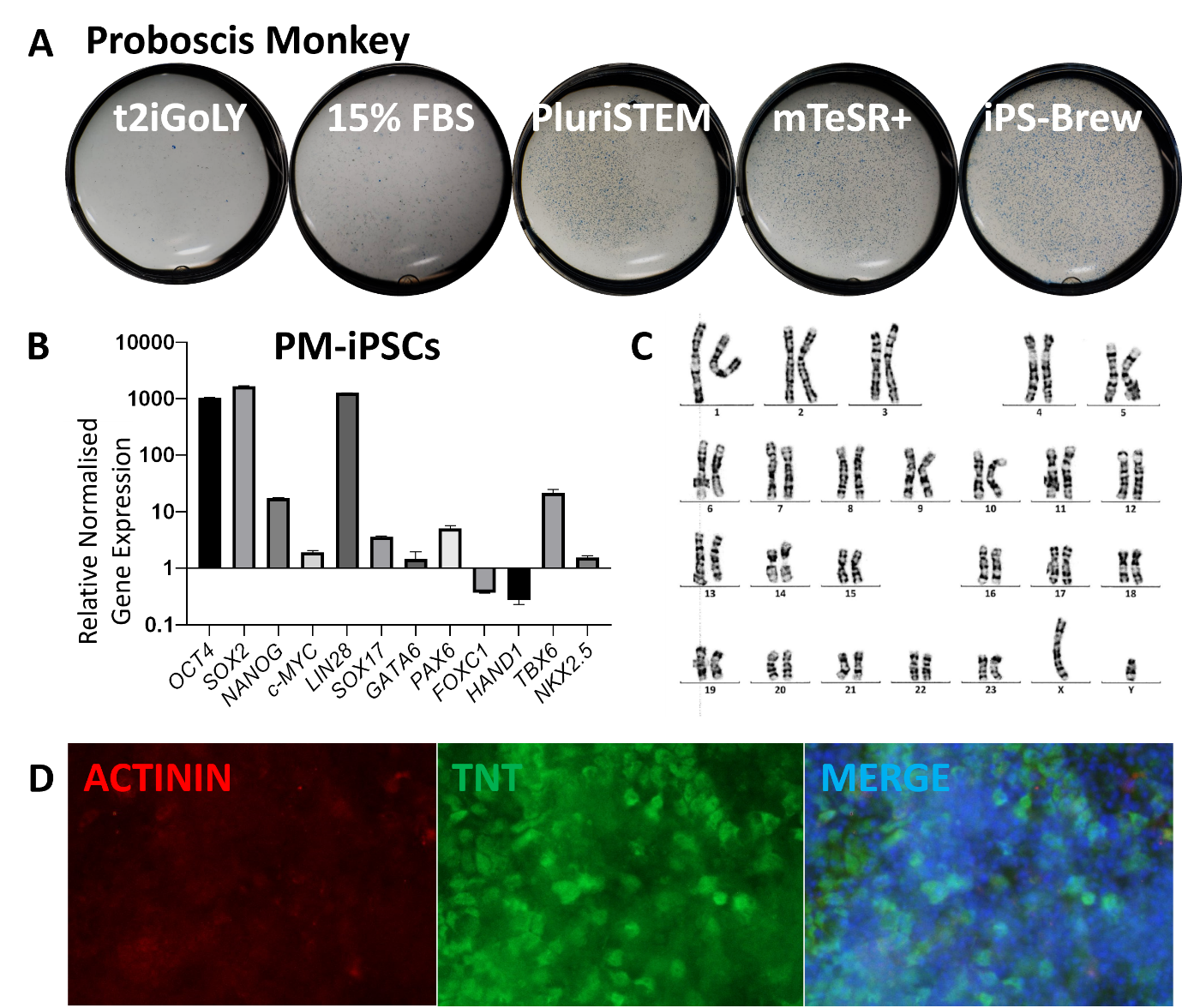
**

**Supplementary Figure 4: Reprogramming for and differentiation of PM-iPSCs**. **A.** AP staining of PM-iPSCs reprogrammed in different media. **B.** Quantitative RT-PCR analysis for the expression of pluripotency and germ layer markers in PM-iPSCs normalized to *GAPDH* and controlled to source PM fibroblasts. **C.** Karyotype of PM fibroblasts demonstrating 48 chromosomes, XY. **D.** ICC staining of PM cardiomyocytes.

**Supplementary Table 1: Primers used in qPCR**

| **Gene** | **Forward** | **Reverse** | **Figure** | **Supp Fig** |
| --- | --- | --- | --- | --- |
| *ACTIN* | ATGCAGAAGGAGATTACTGCC | ATCTTGTTTTCTGCGCAAGTTAG | All qPCR | All qPCR |
| *GAPDH* | TTGCCCTCAACGACCACTTT | TGGTCCGGGGGTCTTACTCC | All qPCR | All qPCR |
| *NANOG* | TGAGCTGGTTGCCTCATGTT | CAGAAGACATTTGCAAGGATGGA | 1E |  |
| *NANOG* | CTGAGATGCCTCACACGGAG | TGTTTGCCTTTGGGACTGGT | 2B, 4F, 5F, 6F | 1B, 1C, 2B, 2D, 2G |
| *NANOG* | CTGCTTCTGGAGGTCATATTTCT | CACAAATCACAGGCATAGGTGAA | 4C |  |
| *NANOG* | GCAGGCAACTCACTTTATCCC | AGTCTCCGTGTGAGGCATCT | 5C, 6C |  |
| *SOX2* | CCTGATTCCAGTTTGCCTCTCT | GCTTCAGCTCCGTCTCCATC | 1E |  |
| *SOX2* | CACACTGCCCCTCTCGC | TCCATGCTGTTTCTTACTCTCC | 2B, 4F, 5F, 6F | 1B, 1C, 2B, 2D, 2G |
| *SOX2* | CCCCCAAATTATTCTTCGCCTG | TCCGGGCTGTTTTTCTGGTT | 4C, 5C |  |
| *SOX2* | CCCCAAATTATTCTTCGCCTG | CGGACTGTTCTTCTGGTTGC | 6C |  |
| *OCT4* | CCTTCGCAAGCCCTCATTTC | TAGCCAGGTCCGAGGATCAA | 1E, 4C, 5C, 6C |  |
| *OCT4* | AGTGTGGTTCTGTAACCGGC | CCTGAGAAAGGAGACCCAGC | 2B, 4F, 5F, 6F | 1B, 1C, 2B, 2D, 2G |
| *c-MYC* | GCAGCCGTATTTCTACTGCG | TCCAGATATCCTCGCTGGGC | 2B, 4F, 6F | 1B, 1C, 2B, 2G |
| *c-MYC* | CAGATCAGCAACAACCGAAA | GGCCTTTTCATTGTTTTCCA | 5F | 2D |
| *LIN28* | CCCCCAGTGGACGTCTTTGT | CTCACCCTCCTTCAAGCTCC | 2B, 4F, 5F, 6F | 1B, 1C, 2B, 2D, 2G |
| *SOX17* | CAAGGGCGAGTCCCGTATCC | CGACTTGCCCAGCATCTTGC | 2B, 4F, 5F, 6F | 1B, 1C, 2B, 2D, 2G |
| *GATA6* | AGAAGCGCGTGCCTTCATC | ATAGCAAGTGGTCTGGGCAC | 2B, 4F, 5F, 6F | 1B, 1C, 2B, 2D, 2G |
| *SOX1* | AAAACACTGGAGACGAACGCC | AAGAAAACGCTTTCCGCCTCC | 2B, 4F, 6F | 1B, 1C, 2B, 2G |
| *SOX1* | CAGTACAGCCCCATCTCCAAC | GCGGGCAAGTACATGCTGA | 5F | 2D |
| *PAX6* | GATAACATACCAAGTGTGTCATCAATA | TGCGCCCATCTGTTGC | 2B, 4F, 5F, 6F | 1B, 1C, 2B, 2D, 2G |
| *FOXC1* | AGTAGCTGTCAAATGGCCTTC | TGCCTTGATGGGTTCCTTTGG | 2B, 4F, 6F | 1B, 1C, 2B, 2G |
| *FOXC1* | AGTAGCTGTCAAATGGCCTTC | TGCCTTGATGGGTTCCTTTAG | 5F | 2D |
| *HAND1* | GAGAGCATTAACAGCGCATTCG | CACGTCCATCAGGTAGGCG | 2B, 4F, 5F, 6F | 1B, 1C, 2B, 2D, 2G |
| *TBX6* | AGAACGGCAGAAACTGTAAGAG | ATTCATGAATGTCTCCACCC | 2B, 4F | 1B, 1C, 2B |
| *TBX6* | AGAACGGCAGAAACTGTAAGAG | ATTCACGAATGTCTCCACCC | 5F | 2D |
| *BMP4* | CTAGGTGAGTGTGGCATCCG | CATAGGTCCCTGCAGTAGCG | 2D, 4G, 6F | 2G |
| *BMP4* | AGCTAGGAGCCATTCCGT | AACGACCATCAGCATTCGGT | 5G |  |
| *PDRM14* | GACAGGCTTCGGATCCACAT | TGGAGGCAGTGAACCTCTTAG | 2D, 4G, 6F | 2G |
| *PRDM14* | ACCTCAAGTACACCCCCTGT | AATGTGGATTCGAAGCCGGT | 5G |  |
| *PDRM1* | CGAAGAGAGGAAGCTCTCGG | TGTTAACCGTCTGGCTTCCC | 2D, 4G, 6F | 2G |
| *PRDM1* | AGCCGAGTGGCTAAGGAAAT | TCACGGCAGCACTTTTGTCT | 5G |  |
| *TFAP2C* | TTTCGCACTAACGGGGTCTC | GACTCGCGGATTTCCATTGC | 2D, 4G, 6F | 2G |
| *TFAP2C* | TGCCTATGTCTGTGAAGCCG | TCCGGTCTTGGCTGAGAAGT | 5G |  |
| *GATA4* | GTAGCACTTGGGCGTTTTCC | CTGTGGGAGTCACGTGCAA | 3B |  |
| *GATA4* | TCTACATGAAGCTCCACGGG | TGGGCTTCCGTTTTCTGGTT | 5F |  |
| *NKX2.5* | GCCGAAAAGAAAGAGCTGTGC | ACCAGATCTTGACCTGCGTG | 3B, 4F |  |
| *DDR2* | GCACCCCTGGATGAAAGTCAA | ATGTTCAGCCAAGTGCAGGA | 3B, 4F |  |
| *NODAL* | AGGGCGAGTGTCCTAATCCT | TTCACTGGGGCACAACAAGT | 5F | 2D |
| *NESTIN* | GCGGGCTACTGAAAAGTTCC | GAGGGTCCTGTACGTGGC | 5F | 2D |
| *AMP1* | GGGGCGAAAACTCTCAAGGA | ACCCAGAAACGCTGGTGAAA | 4F, 5F |  |
| *AMP2* | ACTACGATACGGGAGGGCTT | TTCCGGCTGGCTGGTTTATT | 4F, 5F |  |

**Supplementary Table 2: Antibodies used in IF**

| **Antibodies** | **Source / Cat. No.** | **Dilution** |
| --- | --- | --- |
| NANOG | Pepro Tech / 500-P236 | 1:1000 |
| OCT4 | Abcam / ab19857 | 1:200 |
| SOX2 | R&D SYSTEMS / AF2018 | 1:50 |
| SSEA-4 | BD Biosciences / MC813-70 | 1:50 |
| AFP | R&D SYSTEMS / MAB1368 | 1:20 |
| SMA | R&D SYSTEMS / MAB1420 | 1:50 |
| TUJ-1 | R&D SYSTEMS / MAB1195 | 1:100 |
| SOX17 | Abcam / ab224637 | 1:250 |
| cTnT | Abcam / ab45932 | 1:400 |
| ACTININ | Abcam / ab9465 | 1:100 |

**Movie 1:** **CM-iPSCs are capable of differentiation into cardiomyocytes with spontaneous contractility.**
